# Research on spatial carving method of glutenite reservoir based on opacity voxel imaging

**DOI:** 10.1038/s41598-024-63643-2

**Published:** 2024-06-03

**Authors:** Hu Zhao, Zhong-wei Zhang, Hong-wei Yang, Guo-hua Wei

**Affiliations:** 1 Natural Gas Geology Key Laboratory of Sichuan Province, Southwest Petroleum University, Chengdu, 610500 China; 2 School of Geoscisence and Technology, Southwest Petroleum University, Chengdu, 610500 China; 3 Geophysical Exploration Institute, Shengli Oilfield Company, SINOPEC, Dongying, 257000 China

**Keywords:** Opacity, Spatial carving, Voxel imaging, Seismic attributes, Volume attributes fusion, Solid Earth sciences, Energy science and technology

## Abstract

The glutenite reservoir in an exploration area in eastern China is well-developed and holds significant exploration potential as an important oil and gas alternative layer. However, due to the influence of sedimentary characteristics, the glutenite reservoir exhibits strong lateral heterogeneity, significant vertical thickness variations, and low accuracy in reservoir space characterization, which affects the reasonable and effective deployment of development wells. Seismic data contains the three-dimensional spatial characteristics of geological bodies, but how to design a suitable transfer function to extract the nonlinear relationship between seismic data and reservoirs is crucial. At present, the transfer functions are concentrated in low-dimensional or high-dimensional fixed mathematical models, which cannot accurately describe the nonlinear relationship between seismic data and complex reservoirs, resulting in low spatial description accuracy of complex reservoirs. In this regard, this paper first utilizes a fusion method based on probability kernel to fuse seismic attributes such as wave impedance, effective bandwidth, and composite envelope difference. This provide a more intuitive reflection of the distribution characteristics of glutenite reservoirs. Moreover, a hybrid nonlinear transfer function is established to transform the fused attribute cube into an opaque attribute cube. Finally, the illumination model and ray casting method are used to perform voxel imaging of the glutenite reservoirs, brighten the detailed characteristics of reservoir space, and then form a set of methods for ' brightening reservoirs and darkening non-reservoirs ', which improves the spatial engraving accuracy of glutenite reservoirs.

## Introduction

As the exploration and development targets gradually shift from structural oil and gas reservoirs to complex hidden oil and gas reservoirs, it is necessary to more accurately characterize the spatial distribution characteristics of reservoirs, establish a more refined reservoir model, and support the exploration and development of complex oil and gas reservoirs. Seismic data has always played an important role in reservoir characterization as it provides three-dimensional spatial information of geological bodies. Obviously, how to accurately mine the spatial characteristics of reservoir from seismic data is the key to solve the problem. Glutenite reservoir possess the characteristics such as strong concealment, rapid lithology change, complex reservoir connectivity and strong heterogeneity, making it difficult to accurately characterize its spatial characteristics. Therefore, in order to better explore and develop glutenite reservoirs, it is essential to investigate the technology that can accurately characterize their spatial characteristics. Direct volume rendering, as one of the most effective volume data visualization methods for visualizing volume data, involves converting each data point in the volume data into voxels with specific color values and opacity through the transfer function. Subsequently, the changes in light passing through each voxel are analyzed to depict the internal characteristics of the volume data and selectively display or shield some data volume characteristics. Therefore, it is widely used in medical imaging, seismic exploration and other fields.

In the field of seismic exploration, Gerald (1999) used the method of direct volume rendering to convert each data point of 3D seismic data into voxels with specific color and luminosity, thereby achieving the spatial characterization of 3D seismic data^1^. Ropinski et al. (2005) utilized volume rendering technology for three-dimensional visualization of seismic data and proposed the concept of lens. Users can observe the internal characteristics of the data volume of interest through the lens^2^. Zhang (2007) proposed a direct volume rendering method for seismic data based on Shear-Warp in a virtual reality environment, which enabled the stereoscopic display of data volume by combining perspective projection^3^. Li (2008) realized the three-dimensional visualization of seismic data by using the parallel projection-based footprint table method^4^. Qin (2013) proposed a multi-dimensional transfer function setting method based on parallel coordinates. Its application can reduce the dimension of high-dimensional space to the parallel coordinates of two-dimensional space to solve the complex and difficult problem of multi-dimensional transfer function setting^5^. Duan et al. (2013) applied GPU-accelerated ray casting volume rendering method to seismic interpretation, and realized the clear display of horizon spatial distribution characteristics through interactive design transfer function^6^. Chen (2018) analyzed the process of realizing 3D visualization of seismic data using the direct volume rendering method, and realized 3D visualization of seismic data by using Coin3D through ray projection algorithm and histogram-based transfer function^7^. Obviously, the predecessors have used the volume rendering method to realize the spatial characterization of seismic data, but the above authors have used one-dimensional transfer function based on scalar value or high-dimensional transfer function based on fixed mathematical model in volume rendering. Although the overall three-dimensional characterization of seismic data volume has been realized, the nonlinear relationship between seismic data and reservoir has not been accurately characterized, resulting in low accuracy of reservoir spatial description. There is a strong nonlinear relationship between seismic data and reservoir. The design of transfer function holds great significance as they are responsible for classifying voxels and assigning optical attributes (color and opacity) in volume rendering. By adjusting the transfer function, the reservoir and non-reservoir in seismic data volume can be accurately distinguished, and the reservoir can be highlighted, so as to achieve the purpose of ' brightening the reservoir and shielding the non-reservoir '. At present, many transfer function design methods have been proposed. To solve the problem of insufficient classification ability of one-dimensional transfer function, researchers have proposed to increase the gradient^8^, curvature^9^, space^10^, texture^11^, size^12^ and other information to design multi-dimensional transfer function. However, multi-dimensional transfer function has problems such as design complexity and difficulty in understanding. In this regard, researchers have proposed the use of parallel coordinates to reduce multidimensional space into two-dimensional space, aiding in the design of multidimensional functions^13^ or the use of hierarchical clustering^14^, density clustering^15^ and probability model clustering^1^ to achieve adaptive generation of multidimensional transfer functions. However, existing transfer function design methods for this purpose have certain limitations. Some methods lack sufficient classification ability, while others are complex, difficult to comprehend, or have inconvenient user interfaces. Moreover, these methods are primarily designed for medical applications and may not be fully applicable to the field of seismic exploration due to the distinct characteristics of seismic exploration data.

In order to better separate the reservoir and non-reservoir in seismic data volume and accurately describe the spatial distribution characteristics of the reservoir, this paper intends to explore the attribute fusion method based on the probability kernel. The objective is to excavate common characteristics between seismic attributes and highlight the characteristics of the reservoir. Subsequently, a hybrid nonlinear transfer function is established to transform the fused attribute cube into a light-blocking attribute cube. The ray projection method of the illumination model is used for reservoir voxel imaging to shield non-reservoir, and finally complete the characterization of the three-dimensional spatial characteristics of the reservoir.

## Methods

The exploration practice shows that the glutenite reservoir has the characteristics of large lateral thickness variations and strong heterogeneity in physical properties, which affects the subsequent reservoir evaluation and the design of development well location. In this paper, based on the seismic attribute, the probability kernel attribute fusion technology is established to excavate the common characteristics between seismic attributes and further highlight the characteristics of glutenite reservoir.

### An attribute fusion method based on probability kernel

There is a nonlinear relationship between seismic attributes and glutenite reservoirs. It is necessary to carry out volume fusion processing to explore the characteristics of reservoirs between seismic attributes.

**Figure 1:**
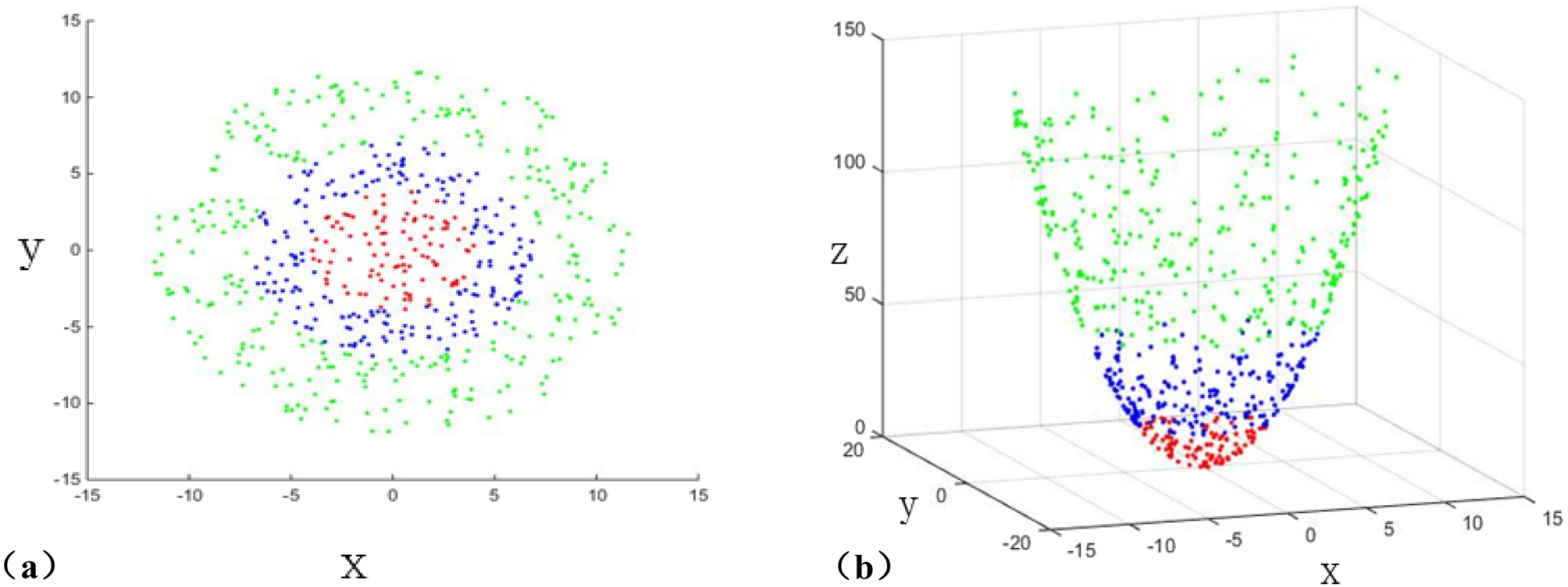
Data nonlinear mapping diagram. (**a**) Seismic attribute data diagram before mapping. (**b**) Seismic attribute data in high-dimensional space after mapping.

1. Firstly, different seismic body attribute data are selected, and the original data of seismic attributes are extracted by the following equation to prepare for fusion :
  1 $$X = \left\{ {x_{1} ,x_{2} ...x_{n} } \right\},x_{i} \in R^{m}$$
  Which $x_{i}$ is the sample point in an individual attribute.
2. Due to the nonlinear relationship between seismic body attributes and glutenite reservoirs, it is not possible to directly indicate reservoir characteristics. Therefore, it is necessary to perform nonlinear mapping on the body attributes involved in the fusion. Firstly, different body attribute data are normalized. On this basis, the kernel function is selected for mapping. The Gaussian kernel function designed in this paper is as follows.
  2 $$K(x_{i} ,x_{j} ) = \exp (\frac{{ - \gamma \left\| {x_{i} - x_{j} } \right\|^{2} }}{{2\sigma^{2} }})$$
  The characteristic of the kernel function is that it has good anti-interference ability to the noise in the data, and can establish a nonlinear relationship between seismic attributes and reservoirs. The above kernel function is used for mapping calculation as follows, $\Phi :x \to \Phi (x) \in R^{f}$.
  3 $$\Phi (X) = \{ \Phi (x_{1} ),\Phi (x_{2} )...\Phi (x_{n} )\} ,\Phi (x_{i} ) \in R^{f}$$
  where : $\Phi (X)$ is the feature space of $X$.
3. Then calculate the eigenvalue $\Lambda$ and eigenvector $V$ of Eq. (3), and arrange them from large to small according to the value, and take the first q eigenvalues with larger eigenvalue contribution rate and the corresponding eigenvectors. The larger the eigenvalue, the stronger the common characteristic.
  4 $$V_{q} = [v_{1} ,v_{2} ,...,v_{q} ]$$
  5 $$\Lambda_{q} = diag(\lambda_{1} ,\lambda_{2} , \ldots ,\lambda_{q} )$$
  where $V$ is the set of eigenvectors $v_{i}$ corresponding to the eigenvalue $\lambda_{{\text{i}}}$, and $\Lambda$ is the diagonal matrix composed of eigenvalues.
4. The feature vector set of high dimensional space is established by using Eqs. (4) and (5).
  6 $$U_{q} = [u_{1} ,u_{2} ,...,u_{q} ] = \Phi JV_{q} \Lambda_{q}^{{ - \frac{1}{2}}}$$
  Here, $U$ is a set of high-dimensional space feature vectors of $u_{i}$, $\Phi$ is the feature space mapped by high-dimensional space, and let $J = N^{{ - \frac{1}{2}}} ({\rm I}_{N} - e{\rm I}^{T} )$, $\Phi J$ be the centralization of high-dimensional feature space data.
5. In order to extract the nonlinear characteristics between the seismic attribute cube more effectively, the Bayesian probability model is used to discard the redundant data. Firstly, the maximum likelihood function estimation parameters are calculated according to the Bayesian probability model using Eq. (7).
  7 $$\begin{gathered} \mu = \Phi_{0} \hfill \\ W = V_{q} (\Lambda_{q} - \rho {\rm I})^{\frac{1}{2}} R \hfill \\ \rho = \frac{1}{n - q}(\sum\limits_{i = q + 1}^{n} {\lambda_{i} } ) \hfill \\ \end{gathered}$$
  And calculate the sample mean:
  8 $$\Phi_{0} = \Phi (X)e,e_{N \times 1} = N^{ - 1} {\rm I}$$
6. Using iterative algorithm to calculate $W$, $\rho$, make it converge :
  9 $$\begin{gathered} Q_{t + 1} = \Sigma Q_{t} (\rho_{t} {\rm I} + M_{t}^{ - 1} Q_{t}^{T} \Sigma Q_{t} )^{ - 1} \hfill \\ \rho_{t + 1} = \frac{1}{n}tr(\Sigma - \Sigma Q_{t} M_{t}^{ - 1} Q_{t + 1}^{T} ) \hfill \\ \end{gathered}$$
  10 $$M = W^{T} W + \sigma^{2} {\rm I}$$
  where $W$ is the load matrix, $Q_{t}$ and $\rho_{t}$ and $Q_{t + 1}$ and $\rho_{t + 1}$ are the empirical load matrix and variance before iteration and the empirical load matrix and variance after iteration, respectively.
7. Finally, the volume fusion calculation is carried out by the following equation.
  11 $$z = R^{T} \Lambda_{q}^{ - 1} R(JQ)^{T} K$$
  where $R$ is an arbitrary orthogonal matrix.
  The key step is Eq. (3). The kernel function is used to map the nonlinear relationship. As shown in Fig. 1a, there are three kinds of seismic attribute data in two-dimensional space, which have a common center, but cannot be effectively separated. The data can be mapped to high-dimensional space by Eq. (3), and the data can be mapped and separated in high-dimensional space and the common center can be identified (Fig. 1b). On this basis, the kernel matrix after mapping is solved, and the feature vector (Eq. 6) of high-dimensional space is established by using Eqs. (4) and (5), and the solution is carried out. Finally, the attribute data fusion calculation (mining the common features between attributes) is completed.

### A volume space characterization method based on opacity voxel imaging

The main idea of seismic opacity voxel imaging is to use the transfer function to map the seismic attributes into the opacity, so as to establish the opacity model and the illumination model, simulate the process of reflection and projection generated by the light penetrating the geological body, and finally calculate the light intensity of each data point in the space. Then the light intensity of each data projected onto the same pixel on the image plane is superimposed to form a visual image. The core problem is the design of the opacity model and the illumination model, which is directly related to the effect of volume rendering.

1. Opacity model design: the opacity model is to convert seismic data into optical impedance value, because seismic data has strong nonlinear characteristics, so the common mathematical functions cannot accurately express the nonlinear relationship between it and the reservoir, in this paper based on the Poisson transfer function (Eq. 12) to construct a hybrid nonlinear opacity model (Fig. 2), which has the characteristics of more concentrated numerical distribution and fast attenuation, which can easily excavate reservoirs from the formation.
  12 $$Q(t) = \frac{{\mathop \lambda \nolimits^{t} \mathop e\nolimits^{ - \lambda } }}{t!}$$

**Figure 2:**
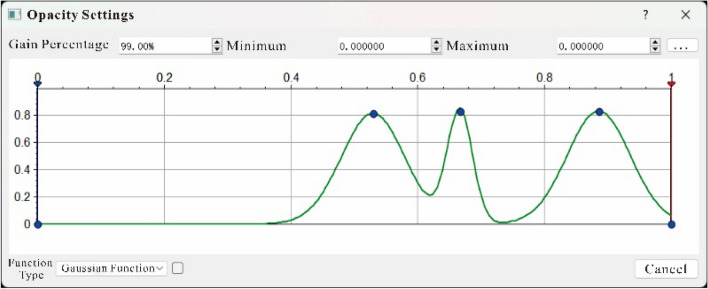
Multi-point Poisson resistance model.
  The smaller the value of $\lambda$ is, the more biased the distribution is. With the increase of $\lambda$, the distribution tends to be symmetrical.
2. Three interpolation volume space resampling calculation ; because the three-dimensional data volume is discretely distributed, the light is transmitted directly, and the screen pixel value can only be calculated (voxel) by relying on the data at the grid points. Therefore, the light may be projected from the gap to the visual plane, resulting in a discontinuous imaging effect of the projected object (Fig. 3),. This requires the resampling calculation of the 3D data field according to the spatial direction of the light penetrating the 3D data volume. This time, the cubic interpolation volume space resampling algorithm is used to solve this problem. The theoretical equation is as follows.
  13 $$f(p) = f(p{}_{2}) + \frac{{y_{p} - y_{p2} }}{{y_{p1} - y_{p2} }}(f(p_{1} ) - f(p_{2} ))$$

**Figure 3:**
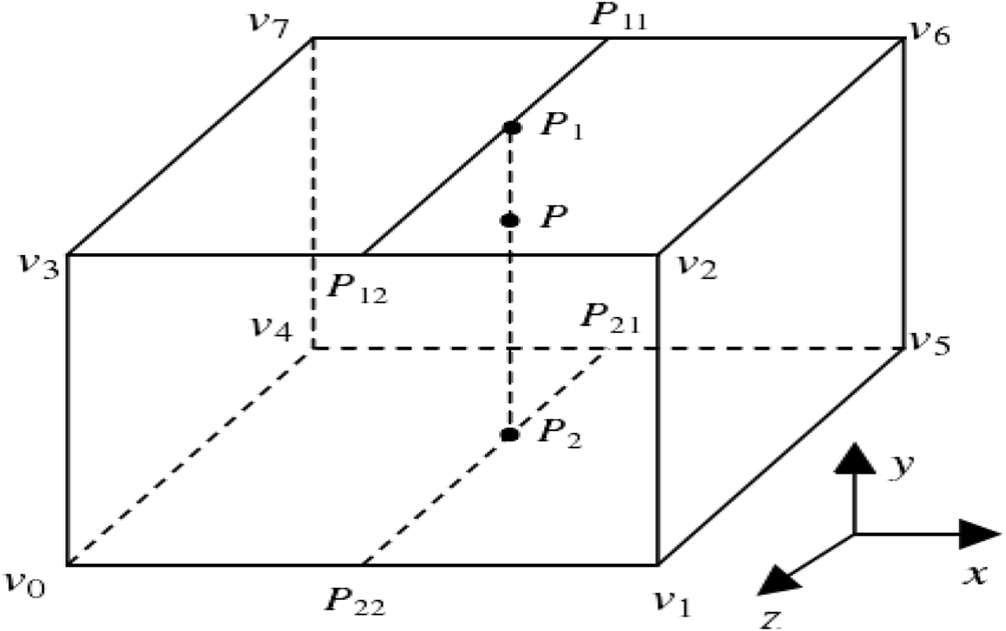
Cubic interpolation volume space resampling schematic diagram.
  Among them, point $p$ is any point in space that needs to be resampled, $p_{1}$ and $p_{2}$ are the points where point $p$ is projected into the data grid, and $y_{p}$ is the $y$ coordinate of point $p$.
3. Lighting model design; In the lighting model, it is assumed that the continuously distributed three-dimensional data field is full of small particles, which can emit light, and the light intensity of each particle reaching the viewpoint is the attenuated light intensity, and the contribution of each particle to the light intensity of the pixel can be accumulated along the line of sight to obtain the final light intensity of the pixel. Then:
  14 $$\frac{dI}{{ds}} = g(s) - \tau (s)I(s)$$
  where $s$ is the light propagation length parameter, $\tau ({\text{s}}) = \rho (s) \cdot A$ is the ray attenuation coefficient, which represents the absorption rate of light by the medium when the ray propagation distance is $s$. $I(s)$ is the light intensity of the incident light.

Move $\tau (s)I(s)$ from the above differential equation to the left of the equation:

15 $$\frac{dI}{{ds}} + \tau (s)I(s) = g(s)$$

Multiply both sides of the equation by ${\text{exp}}(\int_{0}^{s} {\tau (t)dt)}$**:**

16 $$(\frac{dI}{{ds}} + \tau (s)I(s)){\text{exp}}(\int_{0}^{s} {\tau (t)dt)} = g(s){\text{exp}}(\int_{0}^{s} {\tau (t)dt)}$$

Integrating both sides of the above equation from the edge of the 3D volume data (S = 0) to the observer point (S = D) yields:

17 $$I(D)\exp (\int_{0}^{D} {\tau (t)dt)} - I_{0} = \int_{0}^{D} {(g(s){\text{exp}}(\int_{0}^{s} {\tau (t)dt)} )ds}$$

where $I_{0}$ is the initial light intensity of the incident light.

The above equation can be simplified to:

18 $$I(D) = I_{0} T(D) + \int_{0}^{D} {g(s)T^{\prime}(s)ds}$$

The approximate numerical solution of the above equation is as follows:

19 $$\begin{gathered} I(D) = I_{0} \prod\limits_{i = 1}^{n} {\mathop t\nolimits_{i} } + \prod\limits_{i = 1}^{n} {\mathop g\nolimits_{i} } \prod\limits_{j = i + 1}^{n} {\mathop t\nolimits_{j} } \hfill \\ = \mathop g\nolimits_{n} + \mathop t\nolimits_{n} (\mathop g\nolimits_{n - 1} + \mathop t\nolimits_{n - 1} (\mathop g\nolimits_{n - 2} + \mathop t\nolimits_{n - 2} (\mathop g\nolimits_{n - 3} + \ldots + \mathop t\nolimits_{2} (\mathop g\nolimits_{1} + \mathop t\nolimits_{1} \mathop I\nolimits_{0} ) \ldots ))) \hfill \\ \end{gathered}$$

Using the above equation, the light intensity value of the incident light penetrates the object to the observer point can be calculated, and the optical resistance value a corresponding to the propagation distance is usually defined:

20 $$\alpha = 1 - T(s) = 1 - \exp ( - \int_{0}^{s} {\tau (t)dt)}$$

According to the above theory, if the luminous intensity $C$ of three-dimensional particles is constant, or the color value $C$ assigned to similar substances is constant, then after a propagation distance $D$, the light intensity $I(D)$ of the light reaching the viewpoint is:

21 $$I(D) = I_{0} T(D) + C(1 - T(D))$$

In the above equation, $T(D)$ is the transparency of this medium of length $D$, and $I_{0}$ is the intensity of the incoming background light. This equation expresses the combined light intensity of the background light $I_{0}$ and the light source assigned the color value $C$ under the action of transparency $T(D)$. The $\alpha$ of 0 means that the medium is completely transparent, so it is invisible, but objects behind it are visible. It is this idea that excavates the characteristics of sandstone and conglomerate reservoirs in sandstone mudstone, and then completes the volume space carving.

## Application in field data

### Regional geological overview

**Figure 4:**
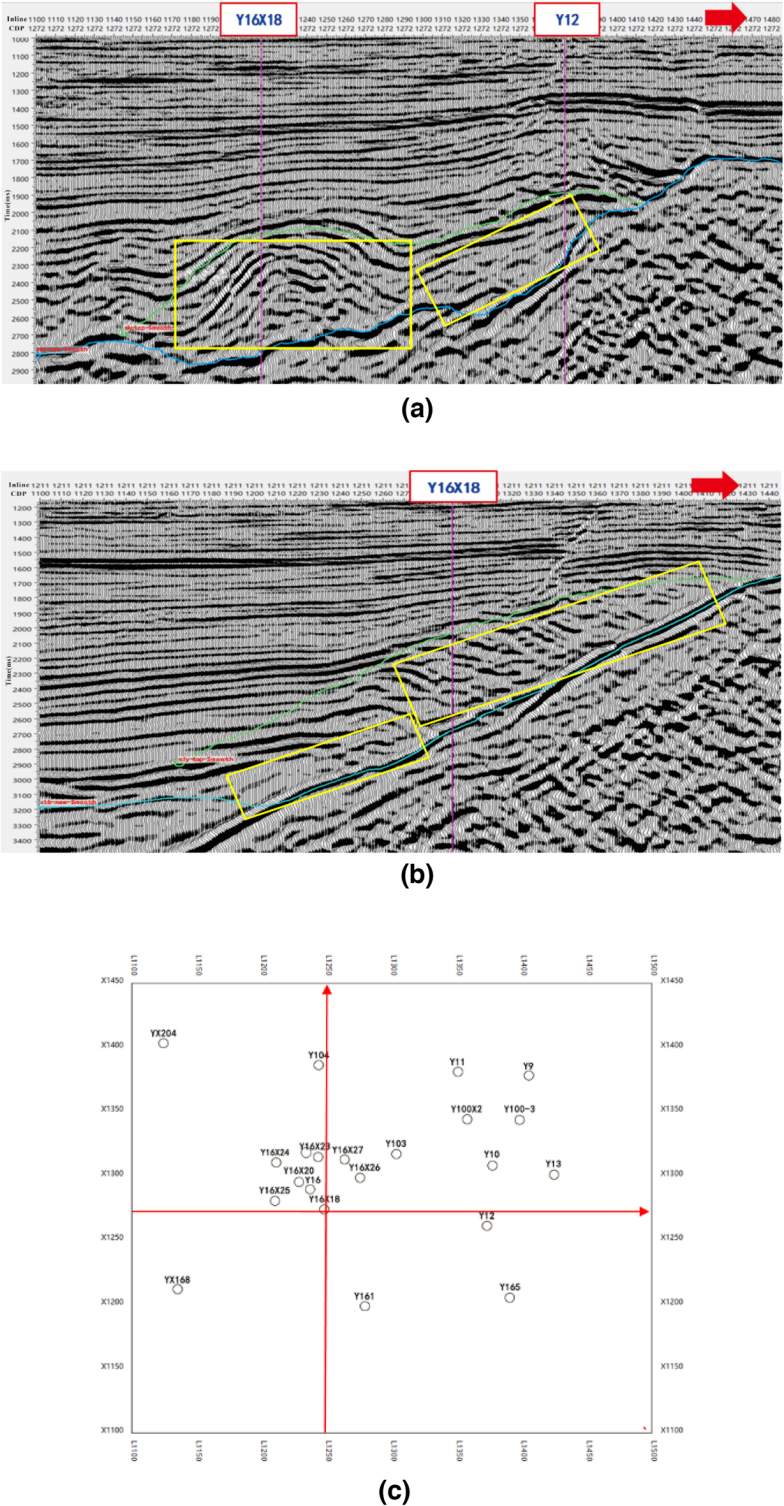
Typical seismic profiles in different directions. (**a**) Seismic profile across wells in east–west direction [the direction of the red arrow in the figure is the direction of the red line arrow parallel to the L number axis of the navigation diagram in (**c**)]. (**b**) Seismic profile across well Y16X18 in north–south direction [the direction of the red arrow in the figure is the direction of the red line arrow parallel to the X number axis of the navigation diagram in (**c**)]. (**c**) Navigation map(X number is CDP number; L number is inline number).

The study area is located in the Yan16 ancient gully in the northern steep slope zone of Dongying Sag in the Jiyang Depression of the Bohai Bay Basin. The main exploration target in this area is the shallow glutenite reservoir, and the oil-bearing area is controlled by the morphology and continuity of the sandstone reservoir. The glutenite primarily represents deposition of a nearshore subaqueous fan system with sediment sources originating from the northern Chenjiazhuang uplift. The lithology is mainly composed of gravel-bearing fine sandstone, interbedded with dark gray and gray mudstone. The reservoir properties are relatively good, but there is a significant variation in spatial continuity, which affects the migration and accumulation of oil and gas. From the Fig. 4, it can be seen that the glutenite reservoir exhibits medium—strong amplitude, bright spots, but with poor continuity in seismic profile. Additionally, the glutenite overlays the basement of the Sinian Formation (This is shown in the yellow box in the figure).

### Application

**Figure 5:**
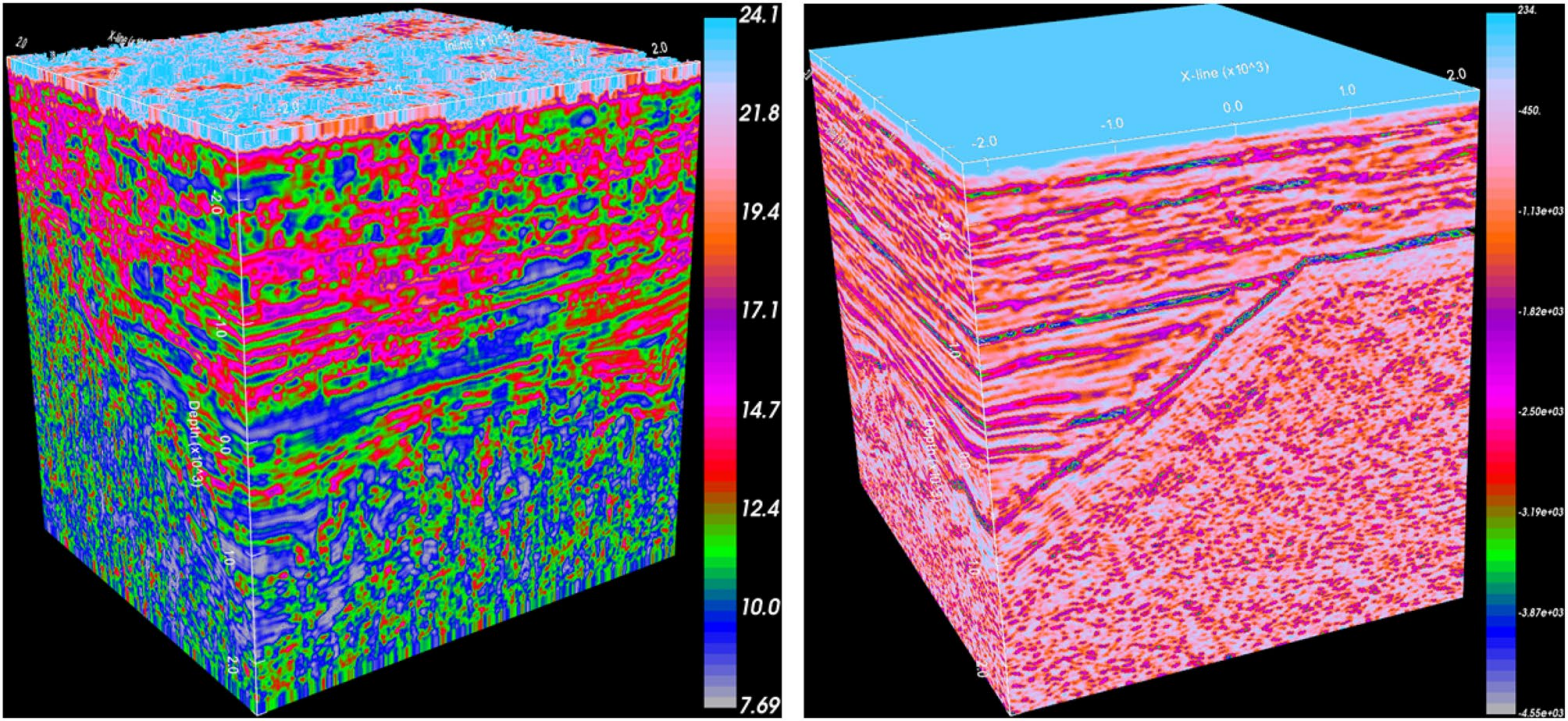
Seismic attribute cube (effective bandwidth on the left, composite envelope difference on the right).

**Figure 6:**
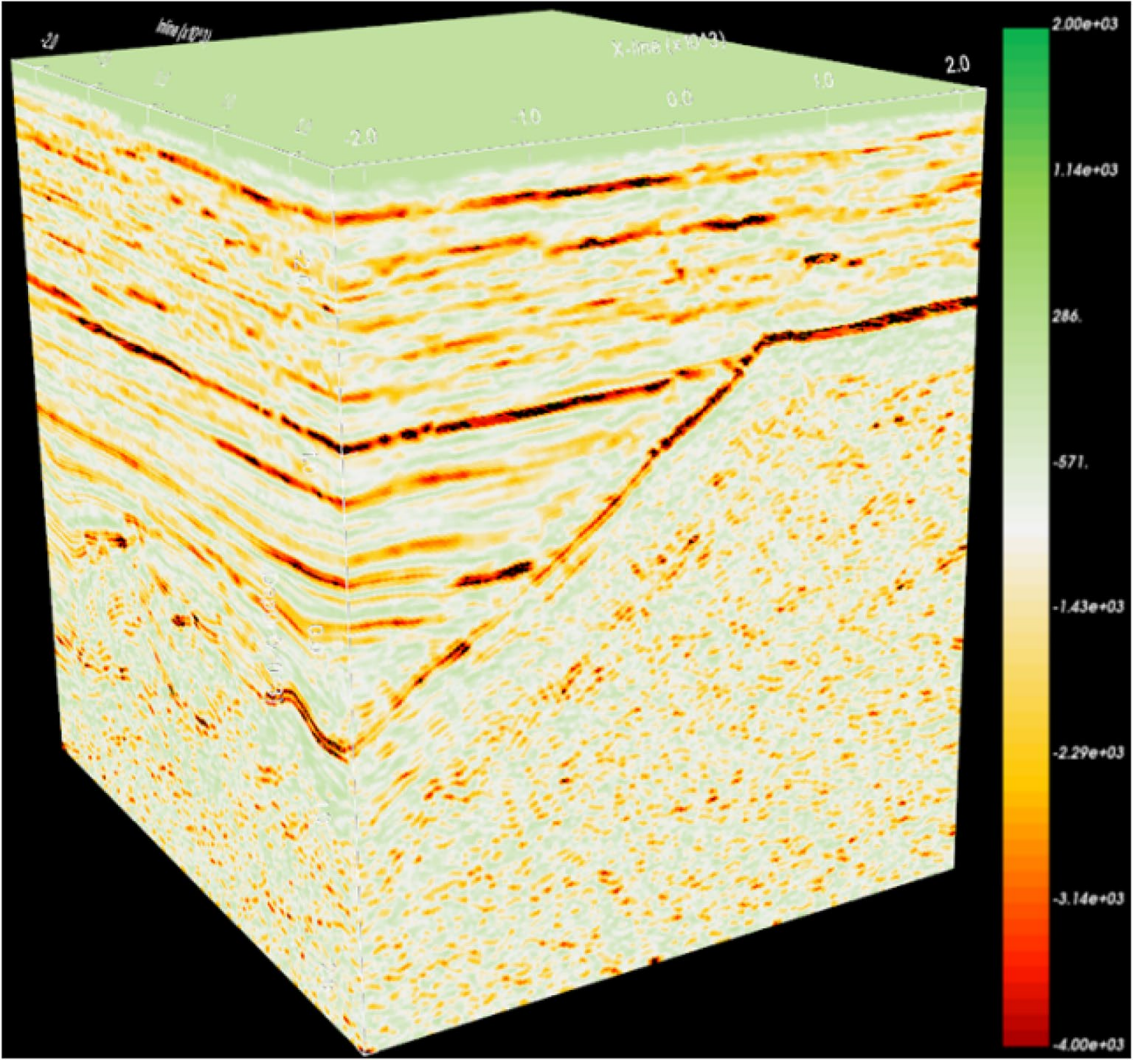
The fused attribute cube.

There is strong lateral heterogeneity in the reservoirs in the study area, for example, well Y16-X19B is effective 11 days after water injection during the development process, while well Y16-X23 at the same distance is indeed ineffective. This clearly indicates a reservoir connectivity issue, highlighting the need for accurately describing the spatial distribution of reservoir. To solve this problem, we first selected sensitive seismic attributes, including effective bandwidth, composite envelope difference and impedance. These attributes can reflect the scale and degree of reservoir development, with higher value indicating more developed reservoirs (Fig. 5). Next, the probabilistic kernel method is used to mine the common features between these attributes. Figure 6 shows the only seismic attribute cube obtained from the fusion process, which reflects the three-dimensional characteristics of all geological bodies in the subsurface. This fused attribute cube serves as the basis and data source of seismic exploration. However, due to the influence of non-reservoirs, the spatial distribution of the glutenite reservoir cannot be fully characterized. Therefore, according to the proposed method in this paper, appropriate transfer function were selected to establish an opacity model, enabling reservoir extraction and non-reservoir masking.

**Figure 7:**
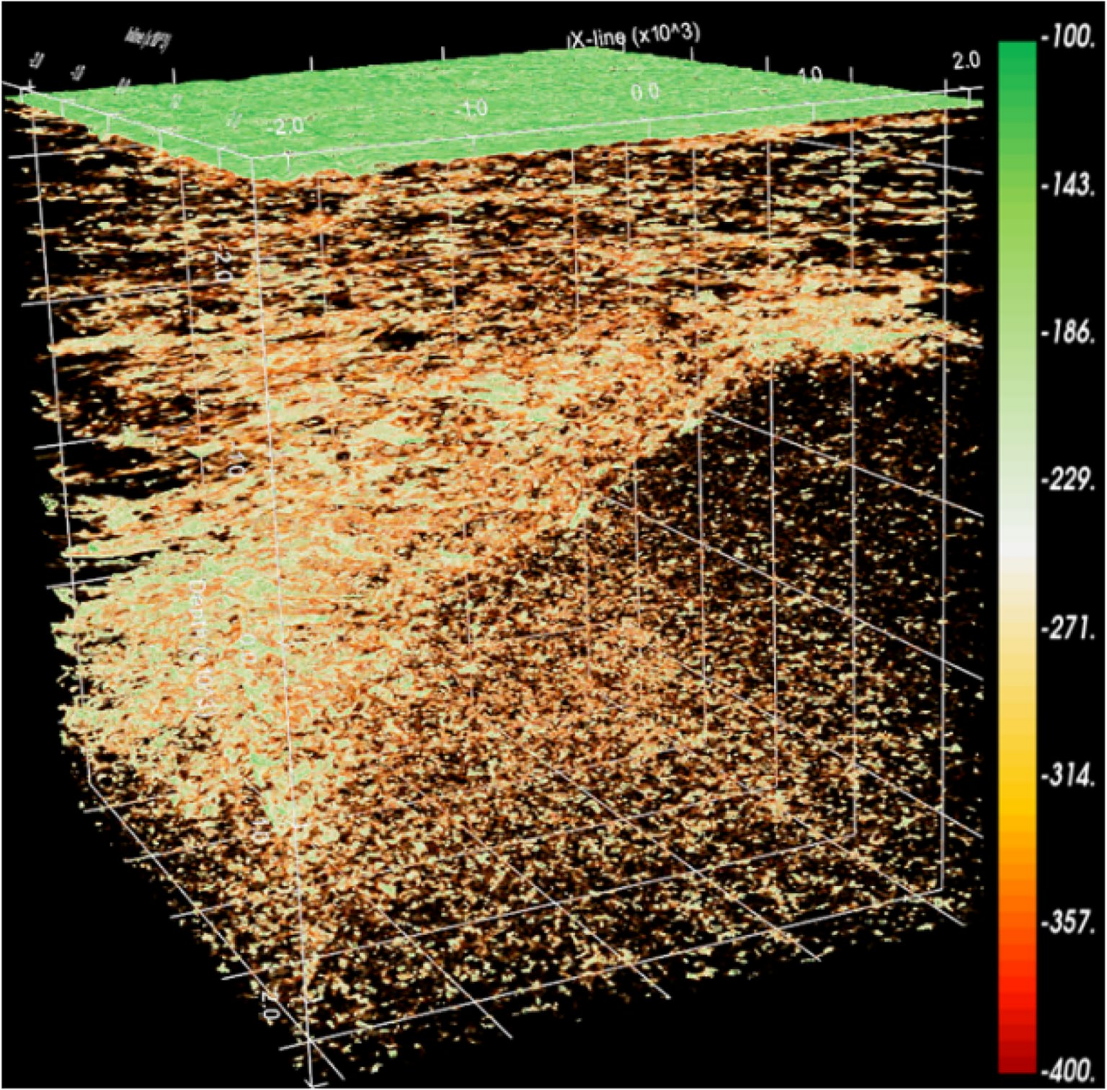
Attribute cube after opacity voxel imaging.

**Figure 8:**
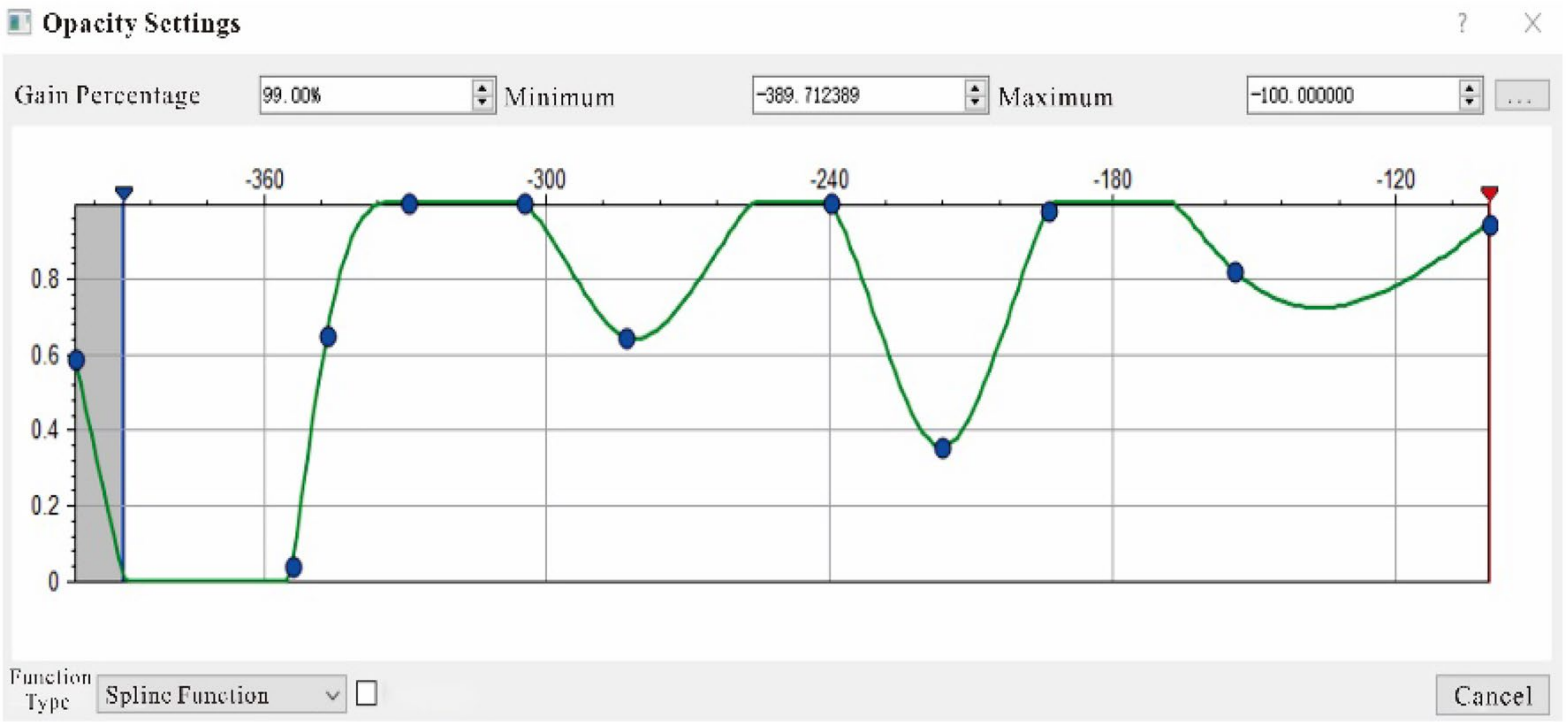
Hybrid multi-segment nonlinear opacity model (the abscissa represents the fused attribute value, and the ordinate represents the opacity value).

**Figure 9:**
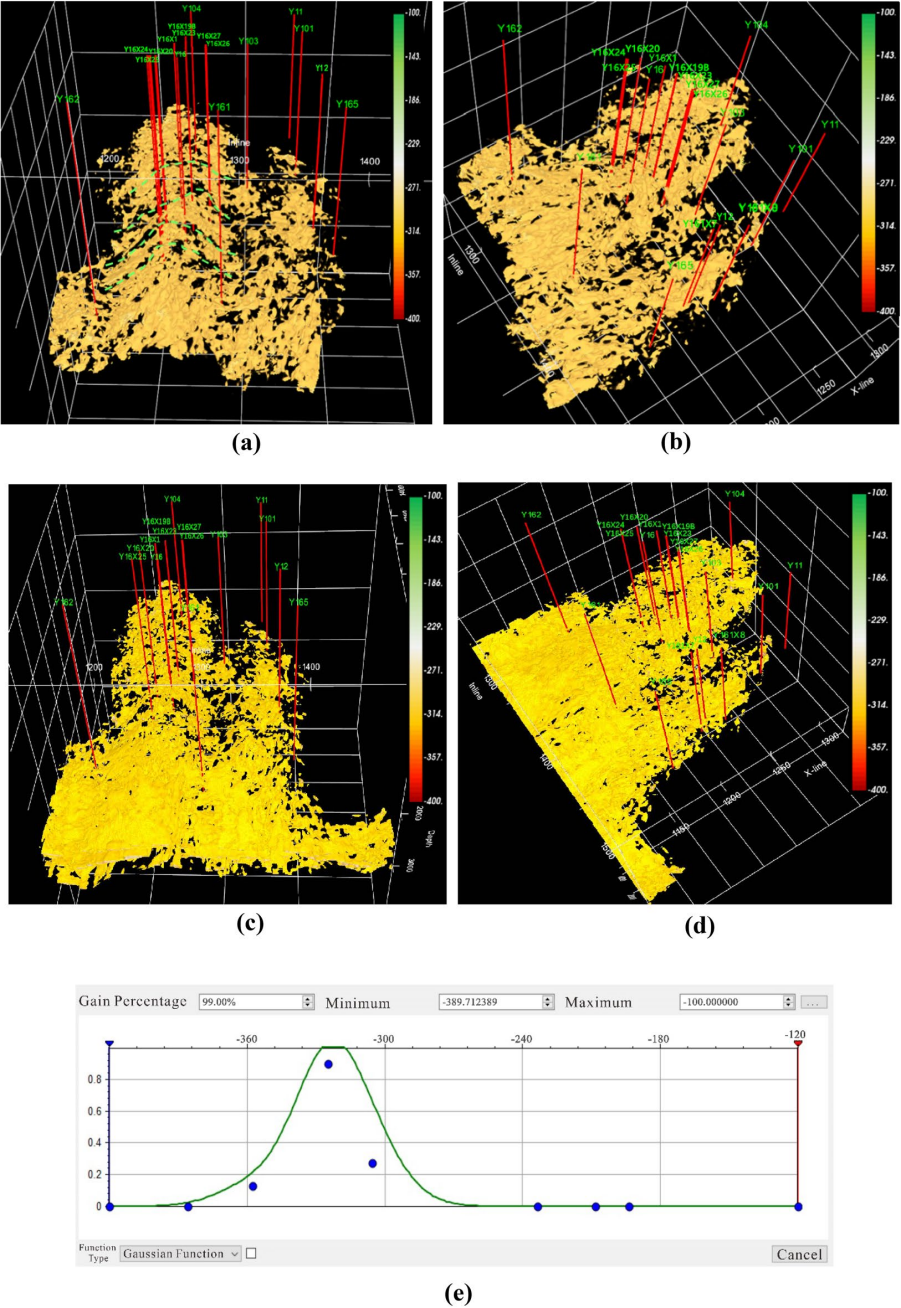
Reservoir characteristics after opacity voxel imaging + target layer surface cropping + volume space rendering. (**a**) Front view, (**b**) oblique view, (**c**) front view (traditional methods), (**d**) oblique view (traditional methods), (**e**) the Gaussian function resistance model (traditional methods).

The traditional method of shielding non-reservoirs is to set a threshold. However, the division boundary between the glutenite reservoir and the non-reservoir is unclear, and the reservoir itself may have multiple ranges of attribute values due to sedimentary processes. Therefore, an opacity model with mixed multi-segment nonlinear transfer function was established according to the characteristics of the glutenite reservoir data (Fig. 8), and corresponding voxel imaging data was obtained(Fig. 7, the more developed the reservoir, the higher the opacity value, with a non-reservoir opacity value of 0). However, in Fig. 7, the spatial features of the glutenite reservoir are still not observable as numerous geological bodies in the non-target layers obstruct the light penetration. To address this, further darkening of the non-reservoir regions was performed. This involved selecting the top and bottom of the target layer for surface cutting, resulting in all geological bodies outside the target layer becoming darker (Fig. 8). Finally, the reservoir is illuminated and non-reservoir layers are darkened through the application of series of technologies (Fig. 9).

In order to show the detailed characteristics of the reservoir, it is necessary to further brighten the reservoir and perform volume rendering calculations to enhance the degree of brightness at each point within the reservoir. Figure 9a, b shows that the application of opacity voxel imaging technology greatly improve the accuracy of reservoir identification. The spatial distribution characteristics of reservoirs are clearly visible, and the lateral continuity is also intuitively reflected. Figure 9c, d display the glutenite reservoir identified using traditional methods (the Gaussian function resistance model, Fig. 9e). Compared with the identification results in Fig. 9a, the traditional method did not effectively excavate the spatial distribution characteristics of the glutenite reservoir on the south side of the study area. In Fig. 9c, the glutenite reservoir on the south side of the study area presents sheet-like characteristics and lacks changes in horizontal heterogeneity. This does not match the actual geological characteristics. The effectiveness of reservoir identification using hybrid multi-segment nonlinear models surpasses that of traditional methods. On this basis, the glutenite reservoir for opacity voxel imaging can be quantified and output, and the output data only contains the data of reservoir points in space, with non-reservoir points assigned 0 value. This data can be applied to reservoir modeling in the later stage. Obviously, the data obtained from the hybrid multi-segment nonlinear opacity model almost only contains reservoir information, and non-reservoir data has been shielded, so the reservoir model established by it will be more reliable.

**Figure 10:**
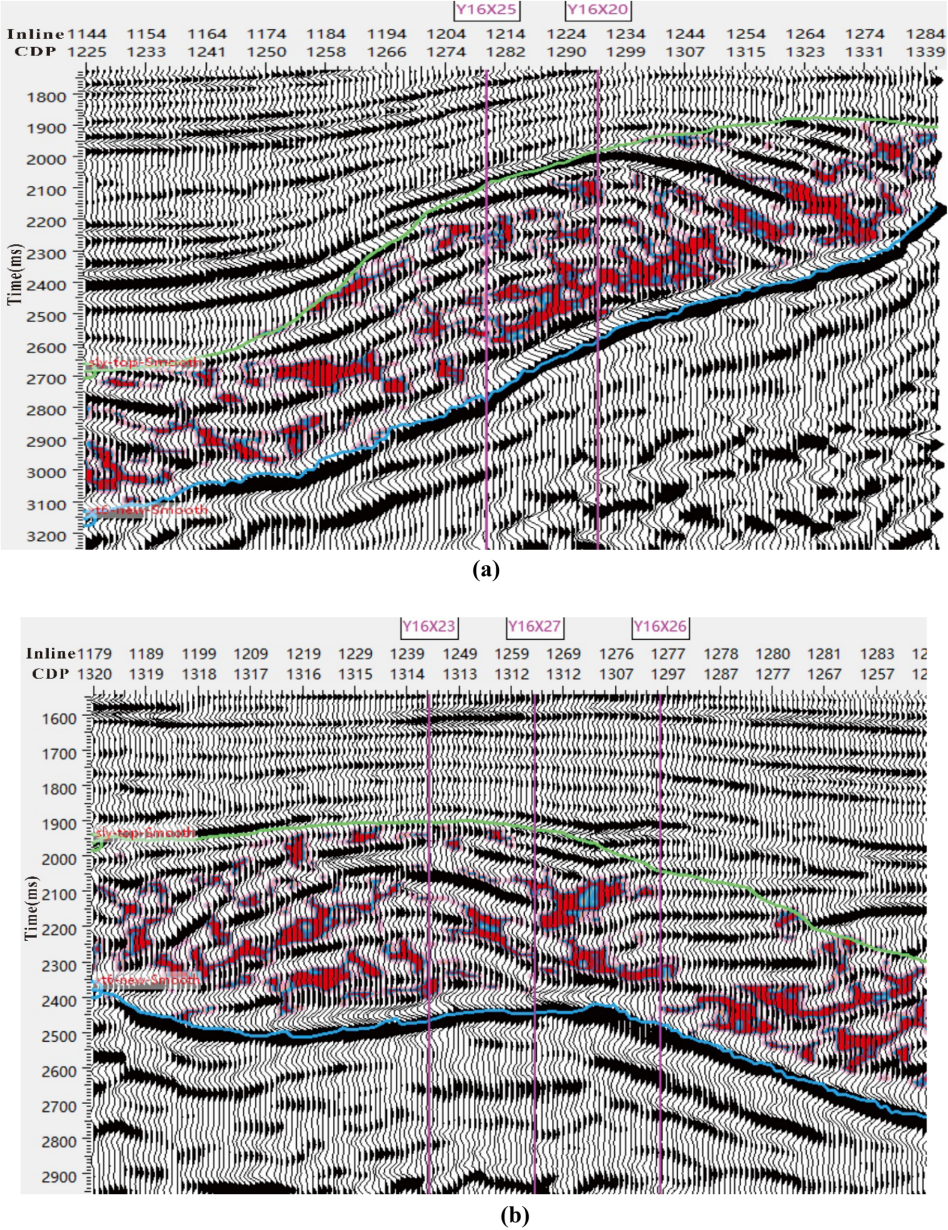
Seismic data, co-rendered with reservoir identified by the proposed method, showing the continuity of reservoir. (**a**) Seismic profile across Well Y16X25. (**b**) Seismic profile across Well Y16X27.

In order to further validate the accuracy of the proposed method, the identified glutenite reservoir was superimposed with seismic data. Figure 9 shows the seismic response characteristics of the glutenite reservoir (red in figure), including medium—strong amplitude, bright spots, discontinuity, and poor continuity. These characteristics are consistent with previous study. In Fig. 10a, it can be seen that the glutenite reservoir between the two wells exhibits good connectivity. This observation aligns with the dynamic production data. Specifically, when water injection occurs in the well Y16X25, there is a significant increase in the water content of the well Y16X20 (Y16X20 has not been injected water). This also supports the reservoir identification results and the actual underground situation. In Fig. 10b, the seismic events between well Y16X27, well Y16X23 and well Y16X26 exhibit good continuity and stable waveform. This suggests that the glutenite reservoir is poorly developed and the mudstone is more developed, so the reservoir connectivity is general. Moreover, the dynamic production data shows that there is no significant response observed between wells Y16X23 and Y16X26 during water injection in well Y16X27, which further supports the reliability of the method of reservoir identification in this paper.

## Conclusions

1. Usually we extract spatial information of reservoir from seismic data and adopt both linear and nonlinear method to establish a mapping relationship between seismic data and reservoirs. However, we rarely investigate whether this function can effectively express the nonlinear relationship between them. Practice suggested that a single low-dimensional or high-dimensional function can not express such a relationship. Therefore, it is necessary to establish a hybrid nonlinear function to accurately express such a nonlinear mapping relationship, enabling an accurate depiction of the volumetric spatial nonlinear relationship between reservoirs and seismic attributes.
2. Opacity voxel imaging technology can intuitively construct multi-segment, multi-type hybrid nonlinear functions, which can map the most favorable seismic information into optical information. This technology demonstrates strong intuitiveness, and the quantification results pertaining to reservoirs align closely with the geological conditions. It significantly enhances the characterization of the spatial distribution characteristics of reservoirs, making the data becomes more reliable for reservoir modeling.

## Data Availability

The data used to support the findings of this study are available from the corresponding author upon request.
